# A Comparative Analysis of Swarm Intelligence Techniques for Feature Selection in Cancer Classification

**DOI:** 10.1155/2014/693831

**Published:** 2014-08-03

**Authors:** Chellamuthu Gunavathi, Kandasamy Premalatha

**Affiliations:** ^1^Department of Computer Science and Engineering, K. S. Rangasamy College of Technology, Tamil Nadu 637 215, India; ^2^Department of Computer Science and Engineering, Bannari Amman Institute of Technology, Tamil Nadu 638 401, India

## Abstract

Feature selection in cancer classification is a central area of research in the field of bioinformatics and used to select the informative genes from thousands of genes of the microarray. The genes are ranked based on *T*-statistics, signal-to-noise ratio (SNR), and *F*-test values. The swarm intelligence (SI) technique finds the informative genes from the top-m ranked genes. These selected genes are used for classification. In this paper the shuffled frog leaping with Lévy flight (SFLLF) is proposed for feature selection. In SFLLF, the Lévy flight is included to avoid premature convergence of shuffled frog leaping (SFL) algorithm. The SI techniques such as particle swarm optimization (PSO), cuckoo search (CS), SFL, and SFLLF are used for feature selection which identifies informative genes for classification. The *k*-nearest neighbour (*k*-NN) technique is used to classify the samples. The proposed work is applied on 10 different benchmark datasets and examined with SI techniques. The experimental results show that the results obtained from *k*-NN classifier through SFLLF feature selection method outperform PSO, CS, and SFL.

## 1. Introduction

Abundant methods and techniques have been proposed for cancer classification using microarray gene expression data. Rapid and recent advances in microarray gene expression technology have facilitated the simultaneous measurement of the expression levels of tens of thousands of genes in a single experiment at a reasonable cost. Gene expression profiling by microarray method has appeared as a capable technique for classification and diagnostic prediction of cancer.

The raw microarray data are images that are transformed into gene expression matrices. The rows in the matrix correspond to genes, and the columns represent samples or trial conditions. The number in each cell signifies the expression level of a particular gene in a particular sample or condition. Expression levels can be absolute or relative. If two rows are similar, it implies that the respective genes are coregulated and perhaps functionally related. By comparing samples, differentially expressed genes can be identified. The major limitation of the gene expression data is its high dimension which contains more numbers of genes and very few samples. A number of gene selection methods have been introduced to select the informative genes for cancer prediction and diagnosis. Feature or gene selection methods remove irrelevant and redundant features to improve classification accuracy. From the microarray data, the informative genes are identified based on their *T*-statistics, SNR, and *F*-test values.

PSO is one of the SI techniques proposed by Kennedy and Eberhart [1] that simulate the behaviour of bird flocking. Yang and Deb [2] proposed the CS inspired by the breeding behaviour of cuckoo. SFL is a memetic metaheuristic that is the combination of two search techniques: the local search of PSO and the competitiveness mixing of the shuffled complex evolution [3]. The randomness in SFL sometimes will not cover an effective area of the search space or it will reflect the same worst solution. To avoid this, the proposed work adopts Lévy flight for position change. The SI techniques such as PSO, CS, SFL, and SFLLF are used for feature selection.

### 1.1. Related Work

In this section the works related to gene selection and cancer classification using microarray gene expression data are discussed. An evolutionary algorithm is used by Jirapech-Umpai and Stuart [4] to identify the near-optimal set of predictive genes that classify the data. Vanichayobon et al. [5] used self-organizing map for clustering cancer data composed of important gene selection step. Rough set concept with dependent degrees was proposed by Wang and Gotoh [6]. In this method they screened a small number of informative single gene and gene pairs on the basis of their dependent degrees.

A swarm intelligence feature selection algorithm was proposed based on the initialization and update of only a subset of particles in the swarm by Martinez et al. [7]. Gene doublets concept was introduced by Chopra et al. [8] based on the gene pair combinations. A new ensemble gene selection method was applied by Liu et al. [9] to choose multiple gene subsets for classification purpose, where the significant degree of gene was measured by conditional mutual information or its normalized form.

A hybrid method was proposed by Chuang et al. [10], which consists of correlation-based feature selection and the Taguchi chaotic binary PSO. Dagliyan et al. [11] proposed a hyperbox enclosure (HBE) method based on mixed integer programming for the classification of some cancer types with a minimal set of predictor genes. The use of single gene was explored to construct classification model by Wang and Simon [12]. This method first identified the genes with the most powerful univariate class discrimination ability and constructed simple classification rules for class prediction using the single gene.

An efficient feature selection approach based on statistically defined effective range of features for every class termed as effective range based gene selection (ERGS) was proposed by Chandra and Gupta [13]. Biomarker identifier (BMI), which identified features with the ability to distinguish between two data groups of interest, was suggested by Lee et al. [14]. Margin influence analysis (MIA) was an approach designed to work with SVM for selecting informative genes by Li et al. [15]. A model for feature selection using signal-to-noise ratio (SNR) ranking was proposed by Mishra and Sahu [16].

Huang et al. [17] presented an improved semisupervised local Fisher discriminant (iSELF) analysis for gene expression data classification. Alonso-González et al. [18] proposed a method that relaxed the maximum accuracy criterion to select the combination of attribute selection and classification algorithm. A quantitative measure based on mutual information that incorporates the information of sample categories to measure the similarity between attributes was proposed by Maji [19]. A feature selection algorithm which divides the genes into subsets to find the informative genes was proposed by Sharma et al. [20].

## 2. Materials and Methods

### 2.1. Gene Selection Methods

#### 2.1.1. *T*-Statistics

Genes, which have considerably different expressions involving normal and tumor tissues, are entrants for selection. A simple *T*-statistic measure given in (1) is used by Yendrapalli et al. [21] to find the degree of gene expression difference between normal and tumor tissues. The top-m genes with the largest *T-*statistic are selected for inclusion in the discriminant analysis. Consider

(1) $$\begin{array}{c} t=\frac{\bar{x_{1}}-\bar{x_{2}}}{\sqrt{v_{1}/n_{1}+v_{2}/n_{2}}}. \end{array}$$

Here $\bar{x_{1}}$: mean of normal samples, $\bar{x_{2}}$: mean of tumor samples, *n*_1_: normal sample size, *n*_2_: tumor sample size, *v*_1_: variance of normal samples, and *v*_2_: variance of tumor samples.

#### 2.1.2. Signal-to-Noise Ratio

An important measure used to find the significance of genes is the Pearson correlation coefficient. According to Golub et al. [22] it is changed to emphasize the “signal-to-noise ratio” in using a gene as a predictor. This predictor is shaped with the purpose of finding the prediction strength of a particular gene by Xiong et al. [23]. The signal-to-noise ratio PS of a gene “*g*” is calculated by

(2) $$\begin{array}{c} \text{PS}\left(g\right)=\frac{\bar{x_{1}}-\bar{x_{2}}}{s_{1}-s_{2}}. \end{array}$$

Here $\bar{x_{1}}$: mean of normal samples, $\bar{x_{2}}$: mean of tumor samples, *s*_1_: standard deviation of normal samples, and *s*_2_: standard deviation of tumor samples.

This value is used to reveal the difference between the classes relative to the standard deviation within the classes. Large values of PS(*g*) indicate a strong correlation between the gene expression and the class distinction, while the sign of PS(*g*) being positive or negative corresponds to *g* being more highly expressed in class 1 or class 2. Genes with large SNR value are informative and are selected for cancer classification.

#### 2.1.3. *F*-Test

*F*-test is the ratio of the variances of the given two sets of values which is used to test if the standard deviations of two populations are equal or if the standard deviation from one population is less than that of another population. In this work two-tailed *F*-test value is used to check the variances of normal samples and tumor samples. Formula to calculate the *F*-test value of a gene is given in (3). Top-m genes with the smallest *F*-test value are selected for inclusion in the further analysis. Consider

(3) $$\begin{array}{c} F=\frac{v_{1}}{v_{2}}. \end{array}$$

Here *v*_1_: variance of normal samples and *v*_2_: variance of tumor samples.

### 2.2. Swarm Intelligence Techniques

#### 2.2.1. Particle Swarm Optimization

PSO is one of the SI techniques that simulate the behavior of bird flocking. It is a population-based optimization tool, which could be implemented and applied easily to solve various function optimization problems.

In PSO, each single solution is like a “bird” in the search space, which is called a “particle.” All particles have fitness values which are evaluated by the fitness function to be optimized and have velocities which direct the flying of the particles. The particles fly through the problem space by following the particles with the best solutions so far.

The original PSO formulae define each particle as potential solution to a problem in *N*-dimensional space. The position of particle *i* is represented as *X*_*i*_ = (*x*_*i*1  _, *x*_*i*2_,…, *x*_*iN*_).Each particle also maintains a memory of its previous best position, represented as *P*_*i*_ = (*p*_*i*1  _, *p*_*i*2_,…, *p*_*iN*_). A particle in a swarm is moving; hence, it has a velocity, which can be represented as *V*_*i*_ = (*v*_*i*1  _, *v*_*i*2_,…, *v*_*iN*_).

Each particle knows its best value so far (*p*best) and the best value so far in the group (*g*best) among *p*bests. This information is useful to know how the other particles around them have performed. Each particle tries to modify its position using the following information:

- the distance between the current position and *p*best,
- the distance between the current position and *g*best.

This modification can be represented by the concept of velocity. Velocity of each agent can be modified by (4). The inclusion of an inertia weight in the PSO algorithm was first reported by Eberhart and Shi in the literature [24]. Consider

(4) Vid=w×Vid+c1×rand(  )×(Pid−Xid)+c2×rand(  )×(Pgd−Xid),

where *i*: index of the particle, *i* ∈ {1,…, *n*}, *N*: population size, *d*: dimension, *d* ∈ {1,…, *N*}, rand(  ): uniformly distributed random variable between 0 and 1, *V*_*id*_: velocity of particle *i* on dimension *d*, *X*_*id*_: current position of particle *i* on dimension *d*, *c*_1_ determines the relative influence of the cognitive component, self-confidence factor, *c*_2_ determines the relative influence of the social component, swarm confidence factor, *P*_*id*_: personal best or *p*best of particle *i*, *P*_*gd*_: global best or *g*best of the group, and *w*: inertia weight.

The current position that is the searching point in the solution space can be modified by the following equation:

(5) $$\begin{array}{c} X_{id}=X_{id}+V_{id}. \end{array}$$

All swarm particles tend to move towards better positions; hence, the best position (i.e., optimum solution) can eventually be obtained through the combined effort of the whole population. The PSO algorithm is simple, easy to implement, and computationally efficient.

#### 2.2.2. Cuckoo Search

Cuckoo search is an optimization technique developed by Yang and Deb in 2009 based on the brood parasitism of cuckoo species by laying their eggs in the nests of other host birds. If a host bird discovers the eggs which are not their own, it will either throw these foreign eggs away or simply abandon its nest and build a new nest elsewhere. Each egg in a nest represents a solution, and a cuckoo egg represents a new solution. The better new solution (cuckoo) is replaced with a solution which is not so good in the nest. In the simplest form, each nest has one egg. A new solution is generated by Lévy flight. The rules for CS are as follows:

- each cuckoo lays one egg at a time and dumps it in a randomly chosen nest;
- the best nests with high quality of eggs will carry over to the next generations;
- the number of available host nests is fixed, and a host can discover a foreign egg with a probability *p*_*a*_ ∈ [0, 1]. In this case, the host bird can either throw the egg away or abandon the nest so as to build a completely new nest in a new location.

When generating new solutions *x*(*t* + 1) for a cuckoo *i*, a Lévy flight is performed using the following equation:

(6) $$\begin{array}{c} x_{i}\left(t+1\right)=x_{i}\left(t\right)+\alpha ⊕\text{Lévy}\left(\lambda \right). \end{array}$$

The symbol ⊕ is an entrywise multiplication. Basically Lévy flights provide a random walk while their random steps are drawn from a Lévy distribution for large steps given in

(7) $$\begin{array}{c} \text{Lévy}\sim u=t^{-\lambda }. \end{array}$$

This has an infinite variance with an infinite mean. Here the consecutive jumps of a cuckoo essentially form a random walk process which obeys a power-law step-length distribution with a heavy tail.

#### 2.2.3. Shuffled Frog Leaping

SFL is swarm intelligence based subheuristic computation optimization algorithm proposed by Eusuff and Lansey [25] to solve discrete combinatorial optimization problem. A group of frogs leaping in a swamp is considered and the swamp has a number of stones at distinct locations on to which the frogs can leap to find the stone that has the maximum amount of available food. The frogs are allowed to communicate with each other so that they can improve their memes using other's information. An individual frog's position is altered by changing the leaping steps of each frog which improves a meme results.

The search begins with a randomly selected population of frogs covering the entire swamp. The population is partitioned into several parallel groups (memeplexes) that are permitted to evolve independently, to search the space in different directions. Within each memeplex, the frogs are infected by other frog's ideas; hence they experience a memetic evolution.

Memetic evolution progresses the quality of the meme of an individual and enhances the individual frog's performance towards a goal. To ensure that the infection process is competitive, it is required that frogs with better memes (ideas) contribute more to the development of new ideas than frogs with poor ideas. Selecting frogs using a triangular probability distribution provides a competitive advantage to better ideas. During the evolution, the frogs may change their memes using the information from the memeplex best or the best of the entire population. Incremental changes in memotype(s) correspond to a leaping step size and the new meme corresponds to the frog's new position. After an individual frog has improved its position, it is returned to the community. The information gained from a change in position is immediately available to be further improved upon.

After a certain number of memetic evolution time loops, the memeplexes are forced to mix and new memeplexes are formed through a shuffling process. This shuffling enhances the quality of the memes after being infected by frogs from different regions of the swamp. Migration of frogs accelerates the searching procedure sharing their experience in the form of infection and it ensures that the cultural evolution towards any particular interest is free from regional bias.

Here, the population consists of a set of frogs (solutions) that is partitioned into subsets referred to as memeplexes. The different memeplexes are considered to be different cultures of frogs, each performing a local search. Within each memeplex, the individual frogs hold ideas that can be influenced by the ideas of other frogs and evolve through a process of memetic evolution. After a defined number of memetic evolution steps, ideas are passed among memeplexes in a shuffling process. The local search and the shuffling processes continue until defined convergence criteria are satisfied. An initial population of *P* frogs is created randomly. For *S*-dimensional problems (*S* variables), a frog *i* is represented as *X*_*i*_ = (*x*_*i*1  _, *x*_*i*2_,…, *x*_*iS*_). Afterwards, the frogs are sorted in a descending order according to their fitness. Then, the entire population is divided into *m* memeplexes, each containing *n* frogs (*P*_*m*×*n*_). In this process, the first frog goes to the first memeplex, the second frog goes to the second memeplex, frog *m* goes to the *m*th memeplex, frog *m* + 1 goes back to the first memeplex, and so forth. Within each memeplex, the frogs with the best and the worst fitnesses are identified as *X*_*b*_ and *X*_*w*_, respectively. Also, the frog with the global best fitness is identified as *X*_*g*_. Then, a process similar to PSO is applied to improve only the frog with the worst fitness (not all frogs) in each cycle.

#### 2.2.4. Shuffled Frog Leaping with Lévy Flight

A Lévy flight is a random walk in which the steps are defined in terms of the step lengths, which have a certain probability distribution, with the directions of the steps being isotropic and random. Lévy flights model activities that involve a lot of small steps scattered with occasional very large trips. Foraging paths of some deer and albatross are examples for Lévy flights. In the case of foraging paths, this result is sensible because the stopping points of a Lévy flight are fractal and in complex ecosystems the distribution of food is fractal. To avoid spending too much time in such unproductive areas, animals need to develop search strategies that generate a fractal distribution of stopping points. Lévy flights have this property. To improve the searching strategy of frogs and performance of classification in SFL, an additional parameter LF is added. The Pseudocodes 1, 2, 3, and 4 represent the pseudocodes of PSO, CS, SFL and SFLLF.

## 3. Feature Selection Based on Swarm Intelligence Techniques

**Figure 1:**
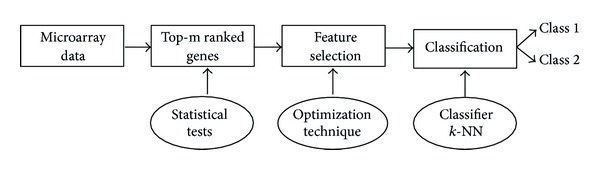
Schematic representation of the proposed method.

The statistical measures are used to identify top-m genes and these genes are further used for feature selection in PSO, CS, SFL, and SFLLF.  Figure 1 gives the schematic representation of the proposed method.

### 3.1. Candidate Solution Representation

**Figure 2:**
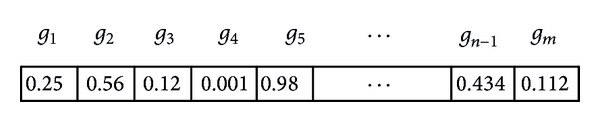
Candidate solution representation.

Figure 2 shows the candidate solution representation of particle position for PSO, egg for CS, and frog for SFL and SFLLF using top-m informative genes which are obtained from statistical techniques. The most used way of encoding the feature selection is a binary string, but the above optimization techniques work well for continuous optimization problem. The random values are generated for gene position. The genes are considered when the value in its position is greater than 0.5; otherwise it is ignored.

### 3.2. Fitness Function

The accuracy of *k*-NN classifier is used as the fitness function [26, 27] for SI techniques. The fitness function fitness(*x*) is defined as

(8) $$\begin{array}{c} \text{fitness}\left(x\right)=\text{Accuracy}\left(x\right). \end{array}$$

Accuracy(*x*) is the test accuracy of testing data *x* in the *k*-NN classifier which is built with the feature subset selection of training data. The classification accuracy of *k*-NN is given by

(9) $$\begin{array}{c} \text{Accuracy}\left(x\right)=\left(\frac{c}{t}\right)\times 100, \end{array}$$

where *c*: samples that are classified correctly in test data by *k*-NN technique and *t*: total number of samples in test data.

## 4. Experimental Setup

In order to assess the performance of the proposed work, ten benchmark datasets are used.  Table 1 shows the datasets collected from Kent Ridge Biomedical Data Repository. The number of samples present in each class is given within parenthesis. The parameters and their values of PSO, CS, SFL, and SFLLF are shown in Table 2.

From the microarray data the discriminative genes are identified and ranked based on *T*-statistics, signal-to-noise ratio, and *F*-test values. The top-m genes are used to represent the candidate solutions of the SI techniques. The values 10, 50, and 100 are assigned to m for testing purpose. The SI technique identifies the features (genes) for classification. The *k*-NN method is used for classification. By empirical analysis the value of *k* is assigned to be 5. The classification accuracy is obtained from 5-fold cross-validation.

## 5. Experimental Results and Discussion

**Figure 3:**
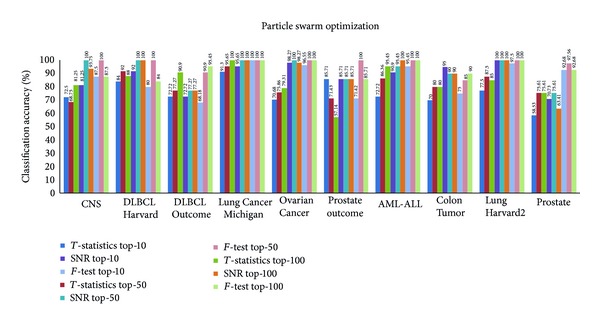
Classification accuracy using particle swarm optimization.

**Figure 4:**
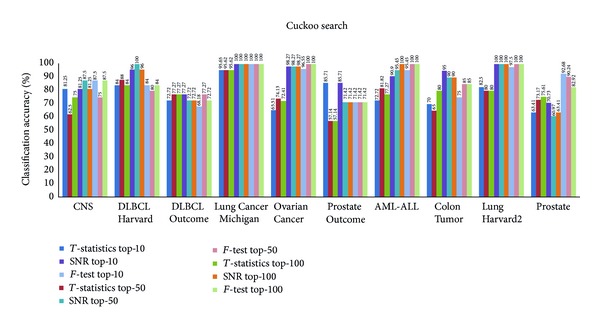
Classification accuracy using cuckoo search.

**Figure 5:**
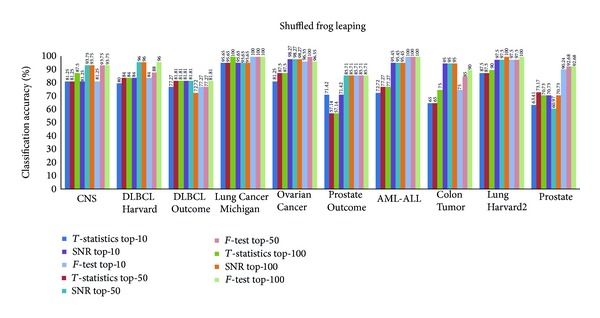
Classification accuracy using shuffled frog leaping.

**Figure 6:**
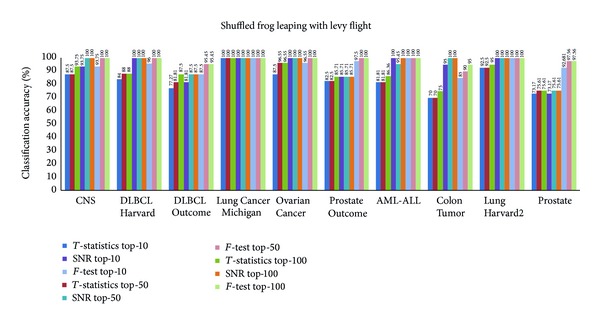
Classification accuracy using shuffled frog leaping with Lévy flight.

Figures 3, 4, 5, and 6 show the results obtained from *k*-NN classifier through the feature selection methods PSO, CS, SFL, and SFLLF, respectively, for top-10, top-50, and top-100 genes obtained from *T*-statistics, SNR, and *F*-test. These results show that for Colon Tumor and Prostate Cancer the 100% accuracy is not achieved by any method. The SFLLF algorithm gives 100% accuracy for Lung Cancer Michigan for all different statistical tests and different numbers of top-m genes. From the results it is inferred that the m value does not influence the accuracy of the classifier. So the value of m should be identified through empirical analysis.

Table 3 compares the maximum classification accuracies obtained from the SI techniques with different statistical measures.

Tables 4, 5, 6, 7, 8, 9, 10, 11, 12, and 13 give the comparison of the proposed work with existing methods. Experimental results show that SFLLF outperforms the existing methods.

## 6. Conclusions

Cancer classification using gene expression data is an important task for addressing the problem of cancer prediction and diagnosis. For an effective and precise classification, investigations of feature selection methods are essential. The swarm intelligence techniques based feature selection methods are simple and can be easily combined with other statistical feature selection methods. It is a simple model based on statistical measures and swarm intelligence techniques that perform two levels of feature selection to get the most informative genes for classification process. *T*-statistics, signal-to-noise ratio, and *F*-test are used to select the important genes that are the reason for cancer. The SI techniques such as PSO, CS, SFL, and SFLLF are applied on the selected top-m genes for feature selection. The *k*-NN is used as a classifier. The experiment results are demonstrated on well-known gene expression benchmark datasets and the performance of SFLLF is compared with PSO, CS, SFL, and the existing works in the literature. The experimental results show that SFLLF outperforms PSO, CS, and SFL. SFLLF feature selection method gives 100% accuracy for 8 datasets out of 10 datasets with *k*-NN classifier.

## Figures and Tables

**Pseudocode 1:**
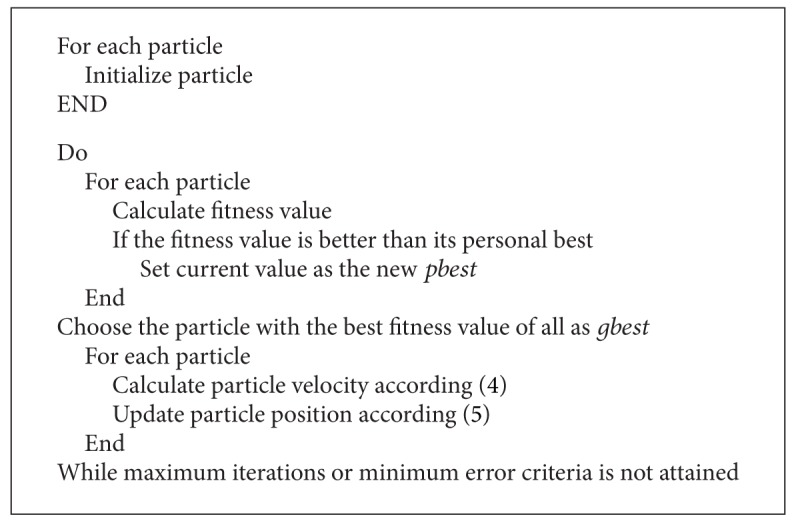
Pseudocode for PSO.

**Pseudocode 2:**
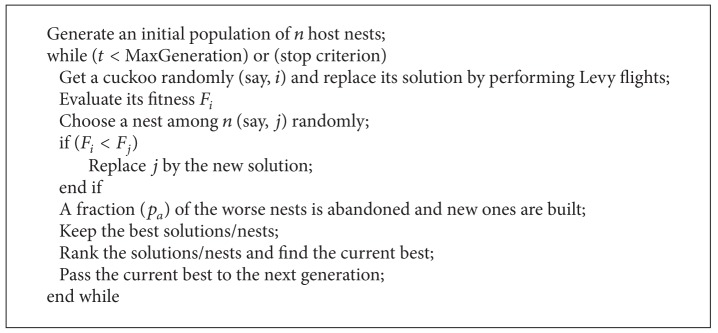
Pseudocode for CS.

**Pseudocode 3:**
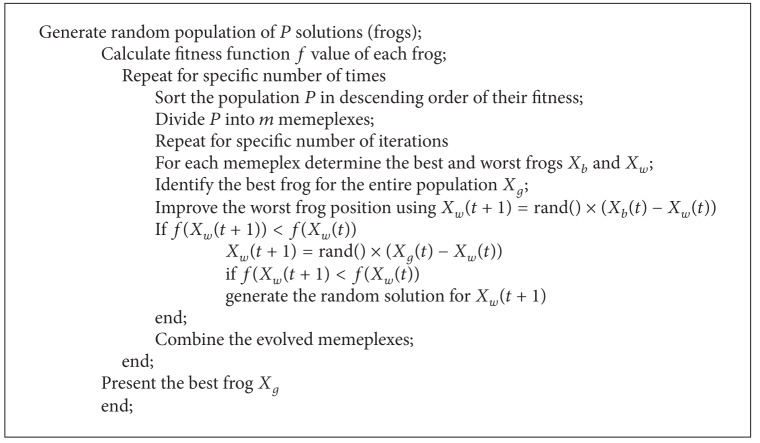
Pseudocode for SFL.

**Pseudocode 4:**
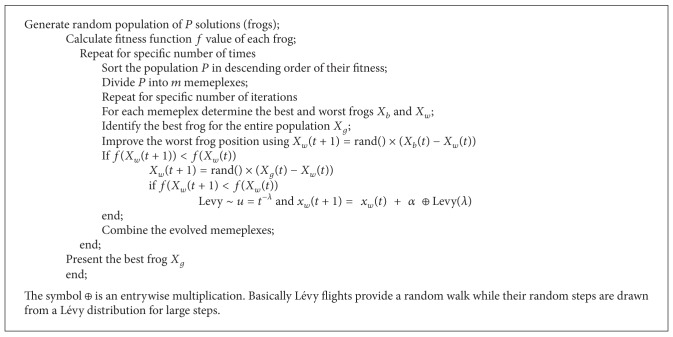
Pseudocode for SFLLF.

**Table 1:** Microarray gene expression datasets.

Dataset name	Number of genes	Class 1	Class 2	Total samples
CNS	7129	Survivors (21)	Failures (39)	60
DLBCL Harvard	7129	DLBCL (58)	FL (19)	77
DLBCL Outcome	7129	Cured (32)	Fatal (26)	58
Lung Cancer Michigan	7129	Tumor (86)	Normal (10)	96
Ovarian Cancer	15154	Normal (91)	Cancer (162)	253
Prostate Outcome	12600	Nonrelapse (13)	Relapse (8)	21
AML-ALL	7129	ALL (47)	AML (25)	72
Colon Tumor	2000	Tumor (40)	Healthy (22)	62
Lung Harvard2	12533	ADCA (150)	Mesothelioma (31)	181
Prostate	12600	Normal (59)	Tumor (77)	136

**Table 2:** Parameters and their values.

Parameter	Value
Particle/egg/frog size	10, 50, 100
Number of memeplexes (*m*)	10
Number of frogs in each memeplex (*n*)	5
Population size	50
Maximum number of generations	200
Shuffling iteration	20
*w*	0.9
*c*_1_	2.1
*c*_2_	2.1
*α*	1
*λ*	1.5
Distance measure in *k*-NN	Euclidean
*k*-value in *k*-NN	5

**Table 3:** Comparison of classification accuracies obtained from different SI techniques.

Dataset name	SI techniques			
	PSO	CS	SFL	SFLLF
CNS	100^~#^	87.5^~#^	93.75^~#^	100^~#^
DLBCL Harvard	100^~#^	100^~^	96^~#^	100^~#^
DLBCL Outcome	95.45^#^	77.27^+~#^	81.81^+~#^	95.45^#^
Lung Cancer Michigan	100^+~#^	100^~#^	100^+#^	100^+~#^
Ovarian Cancer	100^~#^	100^#^	100^#^	100^~#^
Prostate Outcome	100^#^	85.71^+~^	85.71^~#^	100^#^
AML-ALL	100^~#^	100^#^	100^#^	100^~#^
Colon Tumor	95^~^	95^~^	95^~^	100^~^
Lung Harvard2	100^~#^	100^~#^	100^~#^	100^~#^
Prostate	97.56^#^	92.68^#^	92.68^#^	97.56^#^

^+^ *T*-statistics.

^~^SNR.

^#^ *F*-test.

**Table 4:** Comparison of classification accuracy with other methods for CNS.

Reference (year)	Methodology	Maximum classification accuracy in percentage
Alonso-González et al. (2012) [18]	Combination of attribute selection and classification algorithm	75.49
Liu et al. (2010) [9]	EGS (ensemble gene selection) method	98.33
This work	PSO	100
This work	Cuckoo search	87.5
This work	SFL	93.75
This work	SFLLF	100

**Table 5:** Comparison of classification accuracy with other methods for DLBCL Harvard.

Reference (year)	Methodology	Maximum classification accuracy in percentage
Huang et al. (2012) [17]	iSELF (improved semisupervised local Fisher) discriminant analysis	94.67
Alonso-González et al. (2012) [18]	Combination of attribute selection and classification algorithm	100
Dagliyan et al. (2011) [11]	HBE (hyperbox enclosure) method	96.1
Chuang et al. (2011) [10]	Correlation-based feature selection (CFS) and Taguchi genetic algorithm (TGA)	100
Chopra et al. (2010) [8]	Based on gene doublets	98.1
Martinez et al. (2010) [7]	Swarm intelligence feature selection algorithm	100
This work	PSO	100
This work	Cuckoo search	100
This work	SFL	96
This work	SFLLF	100

**Table 6:** Comparison of classification accuracy with other methods for DLBCL Outcome.

Reference (year)	Methodology	Maximum classification accuracy in percentage
Alonso-González et al. (2012) [18]	Combination of attribute selection and classification algorithm	67.84
Wang and Simon (2011) [12]	Univariate class discrimination with single gene	74
This work	PSO	95.45
This work	Cuckoo search	77.27
This work	SFL	81.81
This work	SFLLF	95.45

**Table 7:** Comparison of classification accuracy with other methods for Lung Cancer Michigan.

Reference (year)	Methodology	Maximum classification accuracy in percentage
Alonso-González et al. (2012) [18]	Combination of attribute selection and classification algorithm	100
Liu et al. (2010) [9]	EGS (ensemble gene selection) method	89.58
This work	PSO	100
This work	Cuckoo search	100
This work	SFL	100
This work	SFLLF	100

**Table 8:** Comparison of classification accuracy with other methods for Ovarian Cancer.

Reference (year)	Methodology	Maximum classification accuracy in percentage
Alonso-González et al. (2012) [18]	Combination of attribute selection and classification algorithm	100
This work	PSO	100
This work	Cuckoo search	100
This work	SFL	100
This work	SFLLF	100

**Table 9:** Comparison of classification accuracy with other methods for Prostate Outcome.

Reference (year)	Methodology	Maximum classification accuracy in percentage
Dagliyan et al. (2011) [11]	HBE (hyperbox enclosure) method	95.24
This work	PSO	100
This work	Cuckoo search	85.71
This work	SFL	85.71
This work	SFLLF	100

**Table 10:** Comparison of classification accuracy with other methods for AML-ALL.

Reference (year)	Methodology	Maximum classification accuracy in percentage
Alonso-González et al. (2012) [18]	Combination of attribute selection and classification algorithm	100
Maji (2012) [19]	Mutual Information	100
Chandra and Gupta (2011) [13]	Effective range based gene selection	98.61
Chuang et al. (2011) [10]	Correlation-based feature selection (CFS) and Taguchi genetic algorithm (TGA)	100
Dagliyan et al. (2011) [11]	HBE (hyperbox enclosure) method	100
Martinez et al. (2010) [7]	Swarm intelligence feature selection algorithm	100
Liu et al. (2010) [9]	EGS (ensemble gene selection) method	100
Chopra et al. (2010) [8]	Based on gene doublets	100
Wang and Gotoh (2009) [6]	Rough sets	100
Vanichayobon et al. (2007) [5]	Gene selection step and clustering cancer data by using self-organizing map	100
Jirapech-Umpai and Sturat (2005) [4]	Evolutionary algorithm	98.24
This work	PSO	100
This work	Cuckoo search	100
This work	SFL	100
This work	SFLLF	100

**Table 11:** Comparison of classification accuracy with other methods for Colon Tumor.

Reference (year)	Methodology	Maximum classification accuracy in percentage
Alonso-González et al. (2012) [18]	Combination of attribute selection and classification algorithm	88.41
Maji (2012) [19]	Mutual information	100
Chandra and Gupta (2011) [13]	Effective range based gene selection	83.87
Li et al. (2011) [15]	Margin influence analysis with SVM	100
Chopra et al. (2010) [8]	Based on gene doublets	91.1
This work	PSO	95
This work	Cuckoo search	95
This work	SFL	95
This work	SFLLF	100

**Table 12:** Comparison of classification accuracy with other methods for Lung Harvard2.

Reference (year)	Methodology	Maximum classification accuracy in percentage
Alonso-González et al. (2012) [18]	Combination of attribute selection and classification algorithm	99.63
Chandra and Gupta (2011) [13]	Effective range based gene selection	100
Wang and Simon (2011) [12]	Univariate class discrimination with single gene	99
Chopra et al. (2010) [8]	Based on gene doublets	100
Wang and Gotoh (2009) [6]	Rough sets	97.32
Vanichayobon et al. (2007) [5]	Gene selection step and clustering cancer data by using self-organizing map	100
This work	PSO	100
This work	Cuckoo search	100
This work	SFL	100
This work	SFLLF	100

**Table 13:** Comparison of classification accuracy with other methods for Prostate.

Reference (year)	Methodology	Maximum classification accuracy in percentage
Wang and Gotoh (2009) [6]	Rough sets	91.18
This work	PSO	97.56
This work	Cuckoo search	92.68
This work	SFL	92.68
This work	SFLLF	97.56
